# INDIVIDUAL, FAMILY, AND COMMUNITY FACTORS THAT SHAPE COGNITIVE AGING: AN ECOLOGICAL SYSTEMS APPROACH

**DOI:** 10.1093/geroni/igae098.0506

**Published:** 2024-12-31

**Authors:** Jeremy Hamm, Jacqueline Mogle

**Affiliations:** Chair; Co-Chair

## Abstract

Healthy cognitive aging is shaped in part by individual and social-environmental factors that span systems delineated by social ecological models of lifespan development. This symposium addresses the role of individual- (beliefs, health behaviors), family- (relationship quality), and community-level (neighborhood quality) factors as predictors of normative and non-normative cognitive aging. Taylor et al. use individual-level data from the Midlife in the United States (MIDUS) Study to demonstrate how a composite of control beliefs, purpose in life, and social support prospectively predicts healthier 9-year trajectories of cognitive aging. Hamm et al. use individual-level MIDUS data to document how (health behavior mechanisms) and when in the lifespan (age-moderated pathways) longitudinal changes in control beliefs predict corresponding shifts in cognitive functioning. McGrath et al. investigate novel methods in measuring individual-level indices of health behavior-dependent muscle function (force stability, explosiveness) that can better predict risk of cognitive impairment. Jang et al. use data from the National Study of Daily Experiences (NSDE) to identify the influence of different types of family-level relationships on the experience of daily memory lapses and associated negative affect. Finally, Cerino et al. use NDSE data to document how better neighborhood quality at the community-level is linked to better cognitive functioning, particularly for individuals who lack control over the daily stressors they experience. This symposium thus contributes to a more comprehensive understanding of how healthier cognitive aging is shaped by modifiable beliefs, behaviors, social dynamics, and neighborhood factors that span multiple ecological systems.

